# Supplementary material for: A longitudinal analysis of function annotations of the human proteome reveals consistently high biases
Source: Database (Oxford). 2025 May 7;2025:baaf036. doi: 10.1093/database/baaf036 (PMC12060720; doi:10.1093/database/baaf036)
Supplement: baaf036_Supp [file baaf036_supp.zip › suppl_data/survey_only_annotation_bias_R1_forpub_supp.pdf]

Supplementary Materials for A Longitudinal Analysis of Function Annotations of the Human Proteome Reveals Consistently High Biases

An Phan, Parnal Joshi, Claus Kadelka, Iddo Friedberg

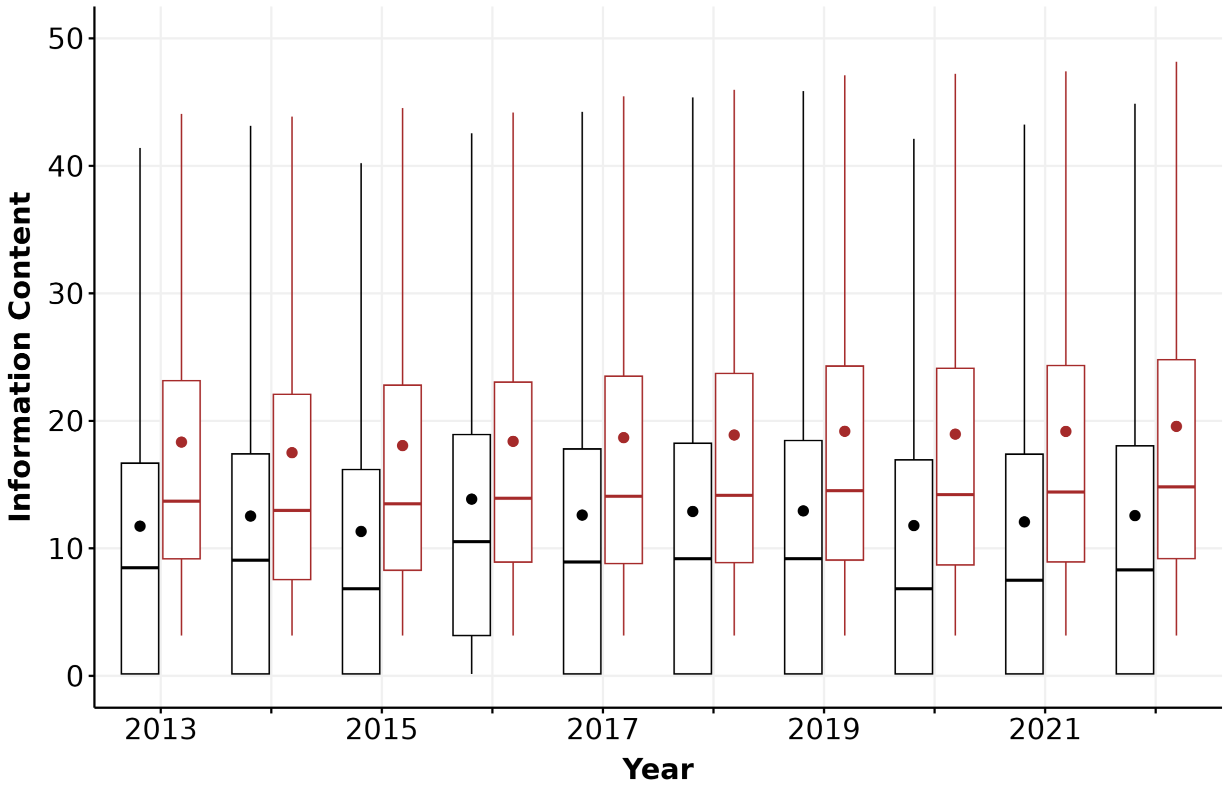

Figure S1: Boxplot showing distribution of information content of all proteins (in black) and the set of proteins excluding protein-binding-only proteins (in brown) in the Molecular function aspect. Each box extends across the interquartile range (IQR), vertical lines extend to the lowest data point (or highest data point) still within 1.5 IQR of the lower quartile (or the upper quartile), a horizontal line shows the median and a dot shows the mean value. Outliers are not displayed.

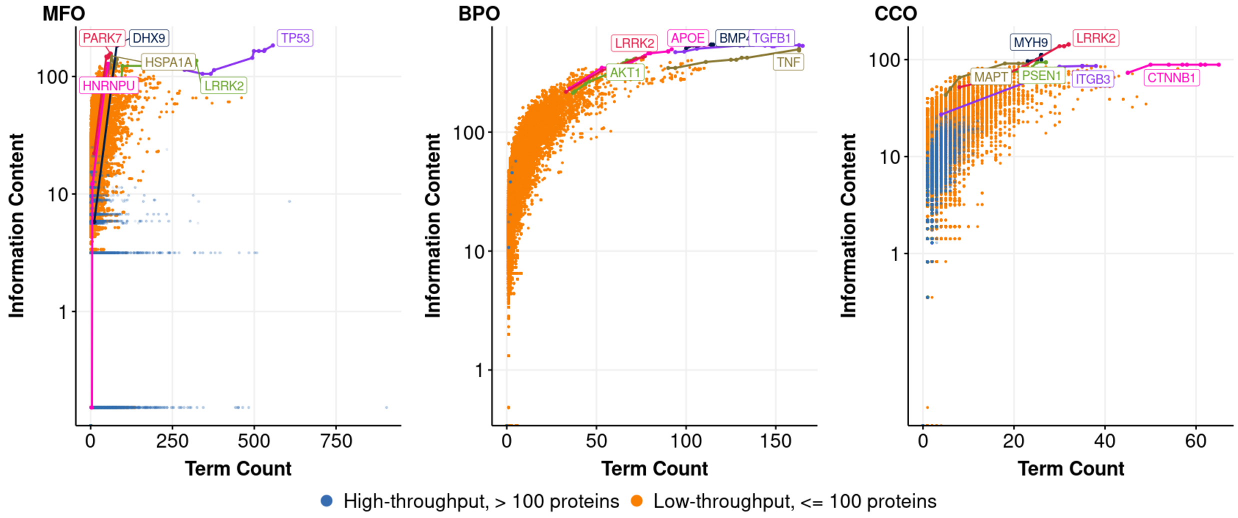

Figure S2: Information content (in log scale) and term count of proteins studied experimentally from 2013 to 2022. Each dot represents the term count and the information content per protein at a time point in Molecular Function (MFO), Biological Process (BPO), and Cellular Component (CCO). Highlighted lines show the growth in information content and unique term count of the most highly annotated proteins over time in low-throughput experiments (gene names encoding for these proteins are labeled).

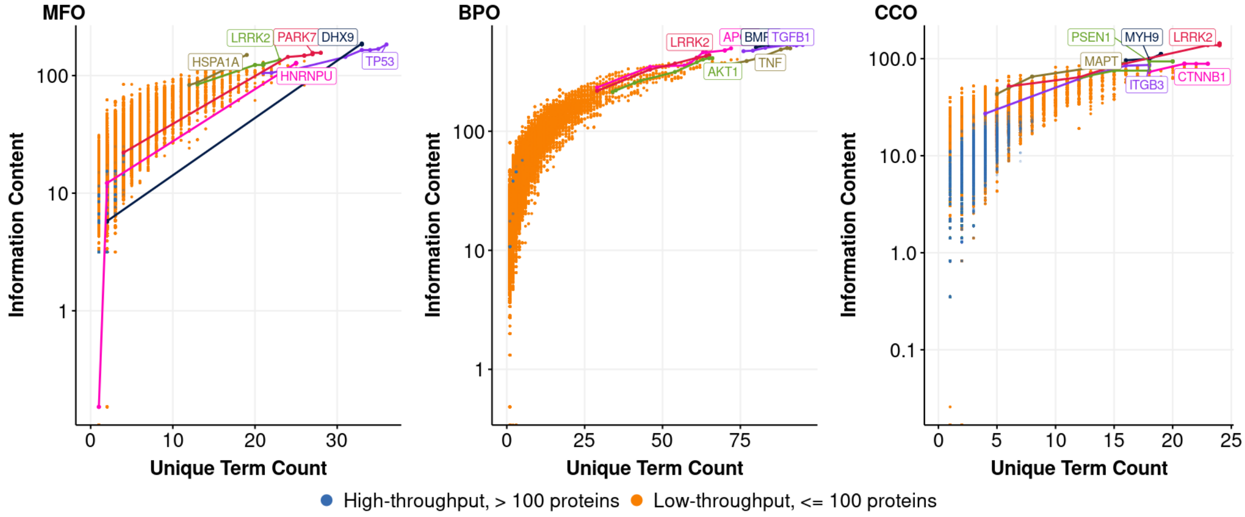

Figure S3: Information content (in log scale) and unique term count of proteins studied experimentally from 2013 to 2022. Each dot represents the term count and the information content per protein at a time point in Molecular Function (MFO), Biological Process (BPO), and Cellular Component (CCO). Highlighted lines show the growth in information content and unique term count of the most highly annotated proteins over time in low-throughput experiments (gene names encoding for these proteins are labeled).

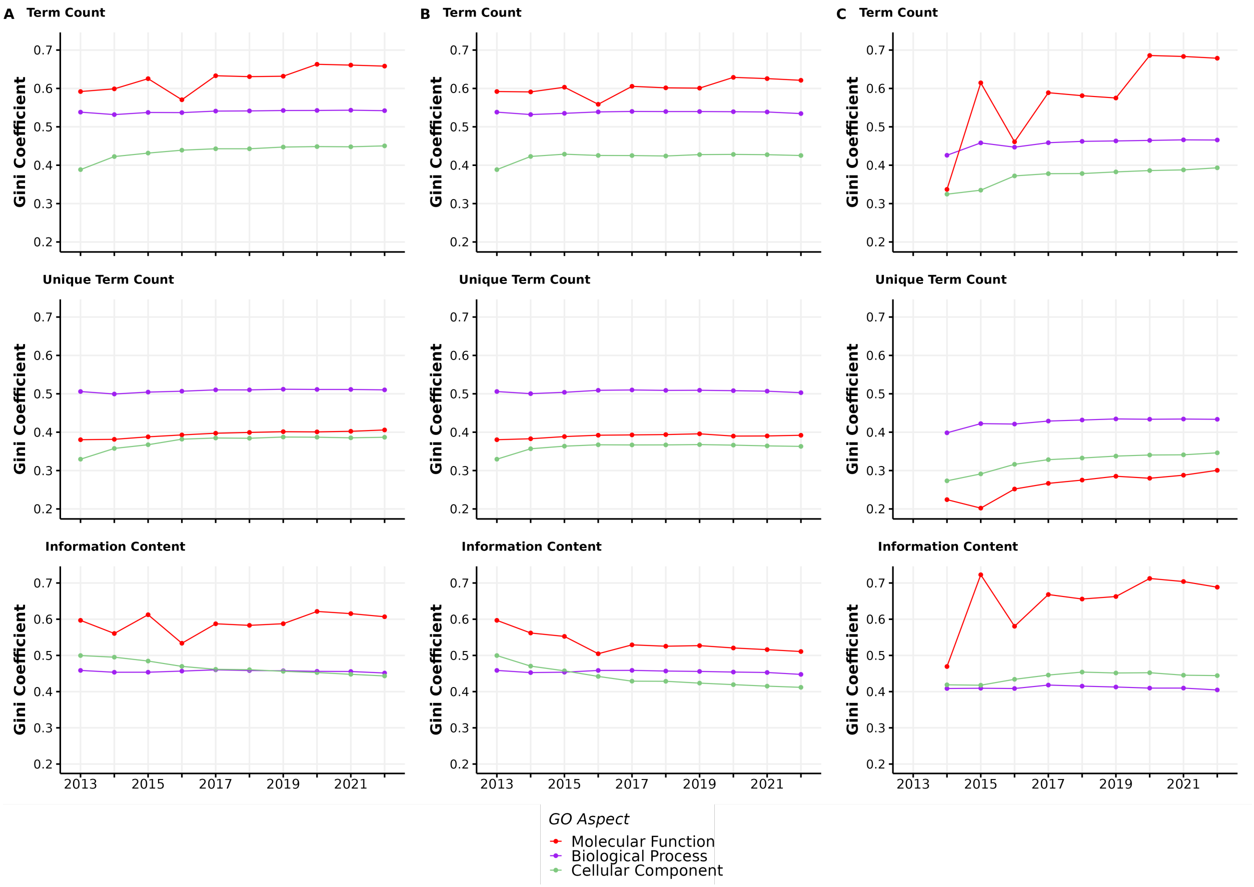

Figure S4: Gini coefficients (0-1) from 2013-2022 calculated from three knowledge metrics (term count, unique term count, and information content) across three GO aspects: Molecular function (MFO, in red), Biological process (BPO, in purple), and Cellular component (CCO, in green). Panel A includes all proteins experimentally annotated in each aspect. Panel B includes proteins annotated in or before 2013, and panel C only includes proteins annotated after 2013.

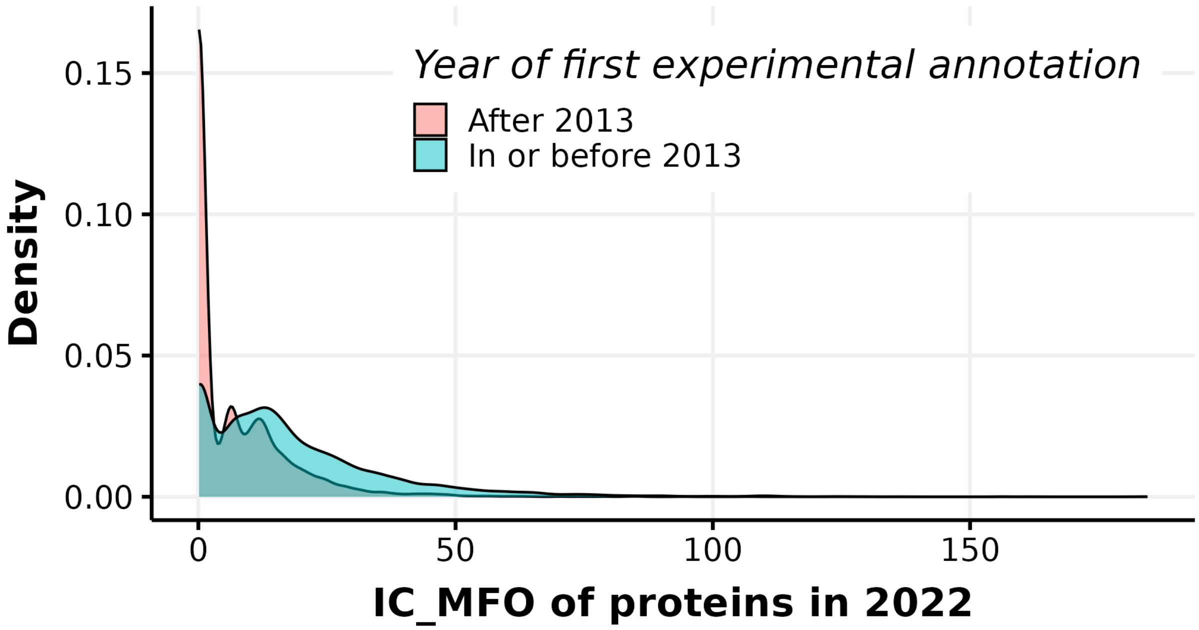

Figure S5: **Density plots showing information content in MFO (IC_MFO) in 2022 of two subsets of proteins: first experimentally annotated in or before 2013, and first experimentally annotated after 2013.**

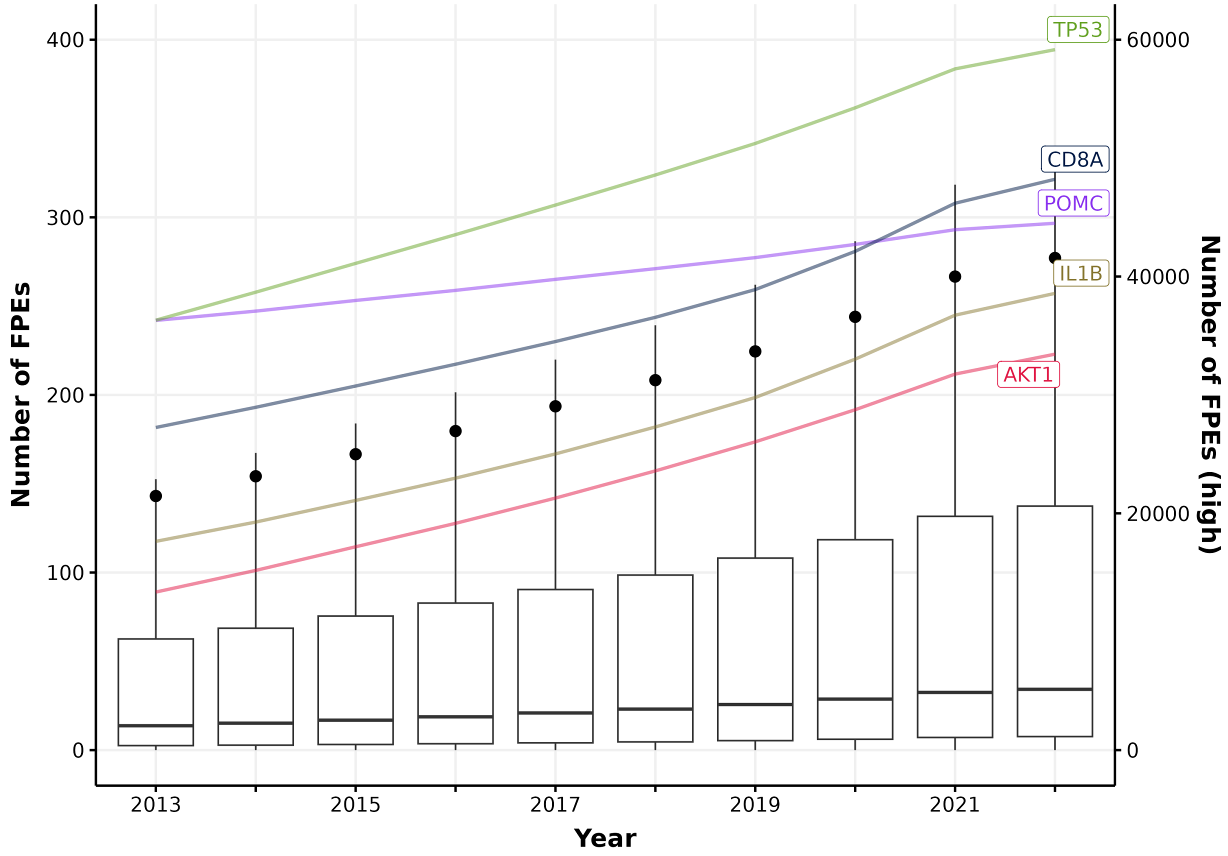

Figure S6: Boxplots showing number of FPEs of proteins from 2013 to 2022. Circles indicate the average number of FPEs of all proteins for each year. Highlighted lines show proteins that have the highest cumulative number of FPEs up to 2022 whose values are shown in the right y-axis of "Number of FPEs (high)". Proteins that are mainly used as clinical assays have been removed.

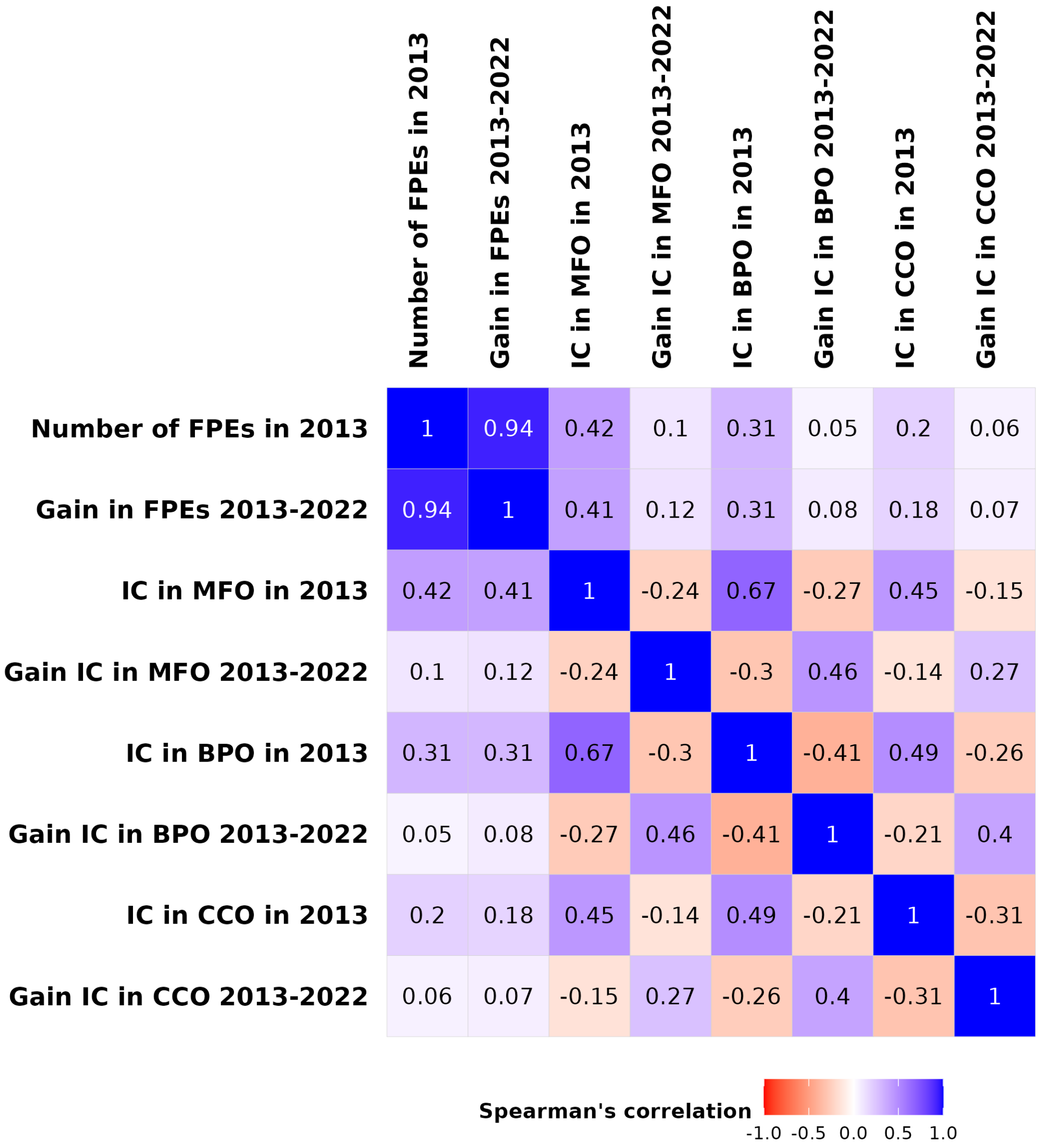

Figure S7: **Heatmap showing Spearman correlation between number of FPEs, information content in every aspect in 2013, and the gain of these metrics from 2013 to 2022.**

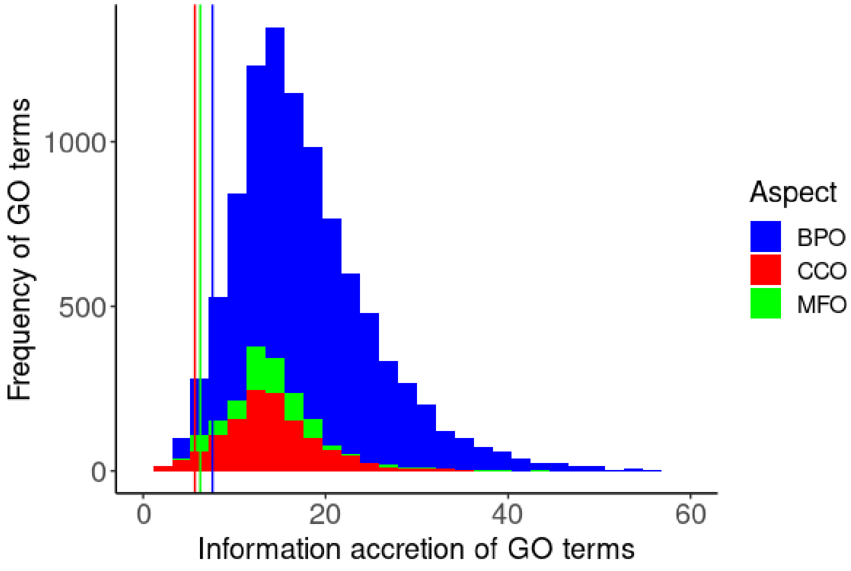

Figure S8: Histogram of information content of GO terms in three aspects of the GO. The vertical lines indicate the 5th percentile, approximately at 6 bits, of the distribution in corresponding color. MFO: Molecular function, BPO: Biological process, CCO: Cellular component.

Table S1. Number of proteins and the median term count and information content by year in all GO aspects. MFO: Molecular function, BPO: Biological process, CCO: Cellular component.

	Number of proteins			Unique term count			Information content		
Year	MFO	BPO	CCO	MFO	BPO	CCO	MFO	BPO	CCO
2013	7831	5856	9023	3	2	2	8.44	24.48	5.74
2014	8953	6887	11348	3	3	2	9.07	25.23	5.74
2015	10946	7598	12469	4	3	2	6.74	26.59	5.96
2016	9414	7619	10056	3	3	2	10.51	26.36	7.94
2017	11747	8393	10865	4	3	3	8.88	27.01	8.6
2018	12000	8651	10913	4	3	3	9.17	27.43	8.94
2019	12253	9041	11193	4	3	3	9.18	27.8	9.16
2020	14238	9304	11340	6	3	3	6.83	28.13	9.45
2021	14335	9436	11466	6	3	3	7.5	28.45	9.5
2022	14544	9869	11791	7	3	3	8.3	29.25	10.27

Table S2. List of proteins with highest information content in at least one GO aspect with UniProtKB identifier, gene name, and protein name.

UniProtKB Identifier	Gene Name	Protein Name
Q08211	DHX9	ATP-dependent RNA helicase A
P02649	APOE	Apolipoprotein E
P12644	BMP4	Bone morphogenetic protein 4
P35222	CTNNB1	Catenin beta-1
P04637	TP53	Cellular tumor antigen p53
P0DMV8	HSPA1A	Heat shock 70 kDa protein 1A
Q00839	HNRNPU	Heterogeneous nuclear ribonucleoprotein U
P05106	ITGB3	Integrin beta-3
Q5S007	LRRK2	Leucine-rich repeat serine/threonine-protein kinase 2
P10636	MAPT	Microtubule-associated protein tau
P35579	MYH9	Myosin-9
Q99497	PARK7	Parkinson disease protein 7
P49768	PSEN1	Presenilin-1
P31749	AKT1	RAC-alpha serine/threonine-protein kinase
P01137	TGFB1	Transforming growth factor beta-1 proprotein
P01375	TNF	Tumor necrosis factor
