# Supplementary figures and images for: A longitudinal analysis of function annotations of the human proteome reveals consistently high biases
Source: Database (Oxford). 2025 May 7;2025:baaf036. doi: 10.1093/database/baaf036 (PMC12060720; doi:10.1093/database/baaf036)

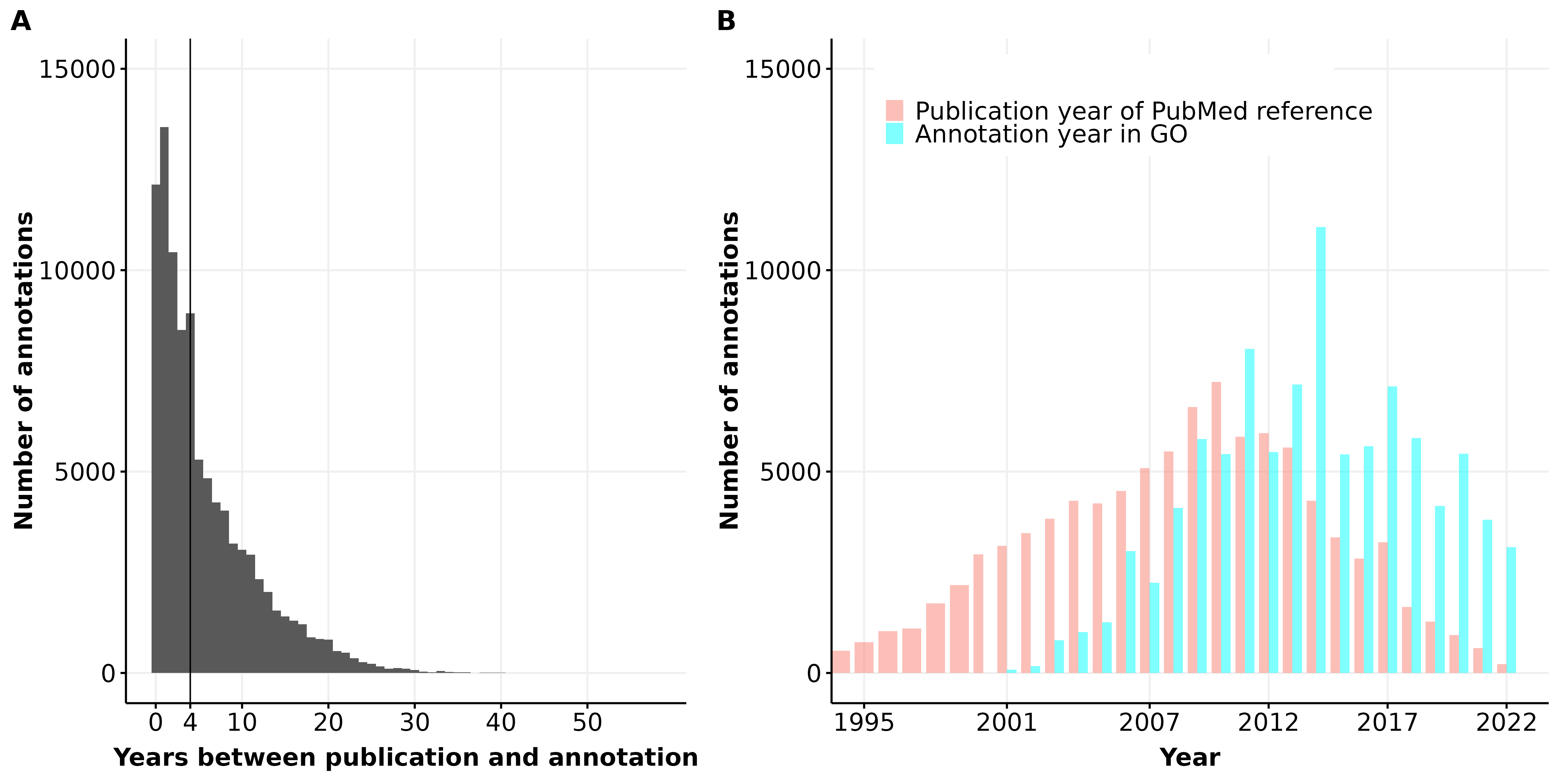

Supplement: baaf036_Supp [file baaf036_supp.zip › suppl_data/delay_curation_comb.png]

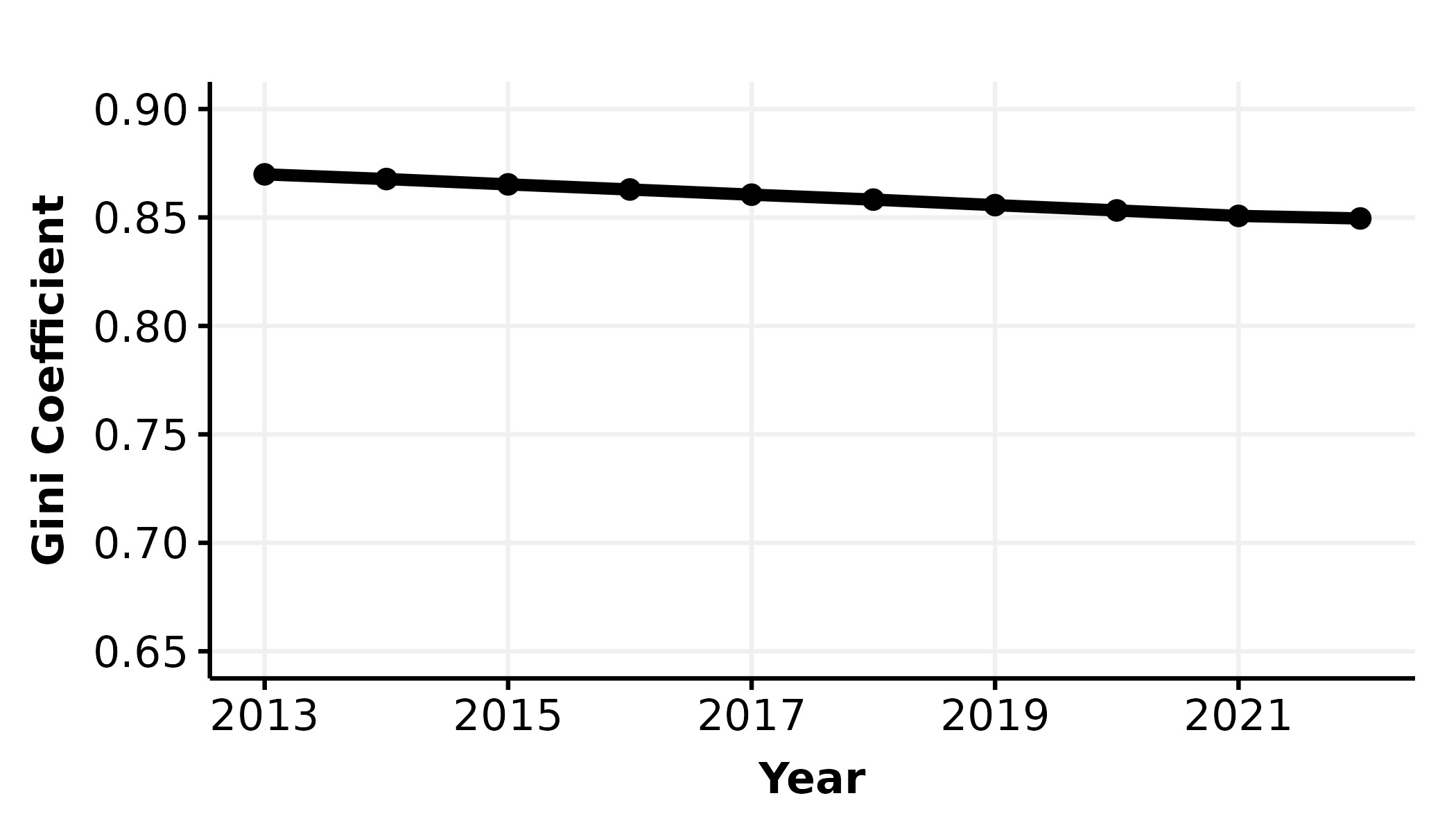

Supplement: baaf036_Supp [file baaf036_supp.zip › suppl_data/gini-ovt-fpe.jpg]

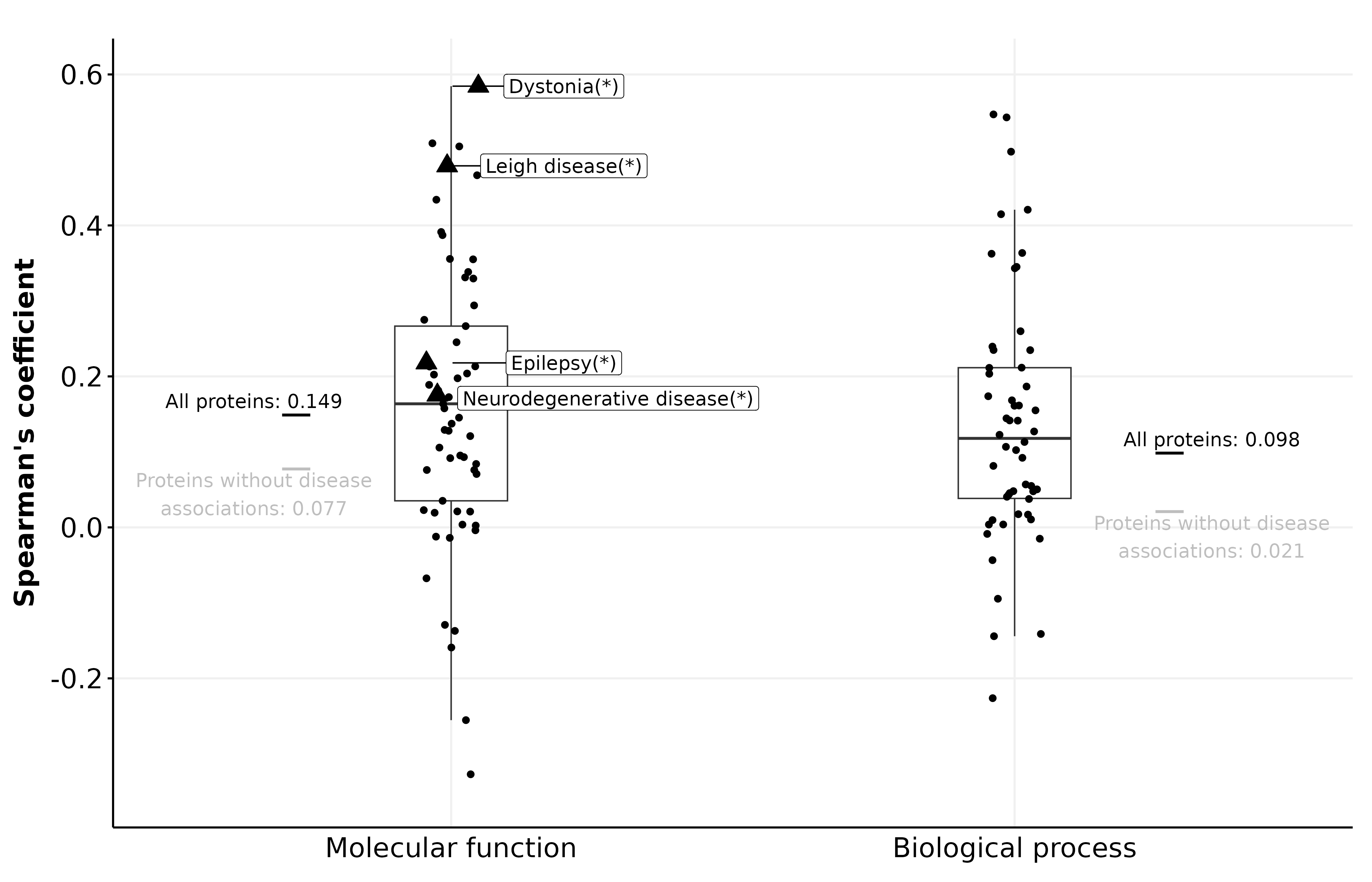

Supplement: baaf036_Supp [file baaf036_supp.zip › suppl_data/scatter-box-diseases-bonferroni.png]

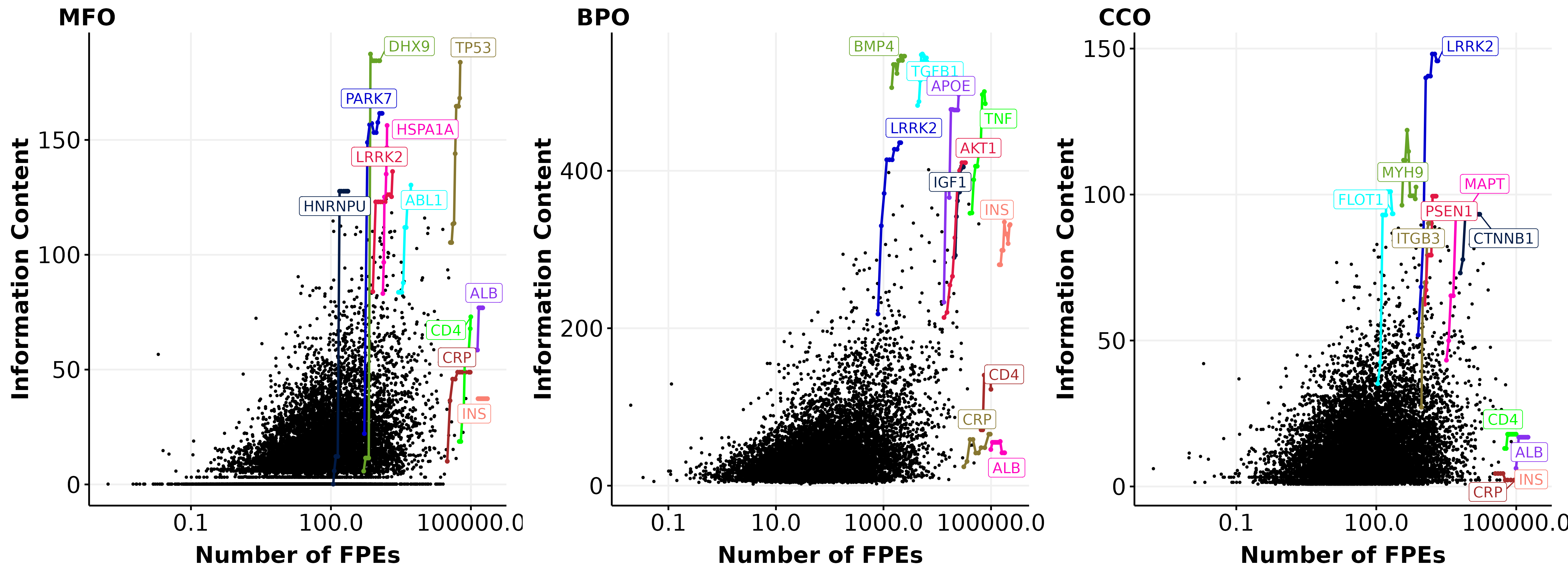

Supplement: baaf036_Supp [file baaf036_supp.zip › suppl_data/scatter-ic-fc_EXP.png]
